# Stink Bug Communication and Signal Detection in a Plant Environment

**DOI:** 10.3390/insects12121058

**Published:** 2021-11-25

**Authors:** Andrej Čokl, Alenka Žunič-Kosi, Nataša Stritih-Peljhan, Maria Carolina Blassioli-Moraes, Raúl Alberto Laumann, Miguel Borges

**Affiliations:** 1 Department of Organisms and Ecosystems Research, National Institute of Biology, SI-1000 Ljubljana, Slovenia; alenka.zunic-kosi@nib.si (A.Ž.-K.); natasa.stritih-peljhan@nib.si (N.S.-P.); 2 Laboratório de Semioquímicos Embrapa Recursos Genéticos e Biotecnologia Brasilia, Brasília 02372, Brazil; carolina.blassioli@embrapa.br (M.C.B.-M.); raul.laumann@embrapa.br (R.A.L.); miguel.borges@embrapa.br (M.B.)

**Keywords:** plant-dwelling insects, biotremology, Pentatominae stink bugs, host plants, evolution, communication, transmission medium, signals, sensory system

## Abstract

**Simple Summary:**

Plant-dwelling stink bugs communicate with chemical and plant-borne vibratory signals that are altered when transmitted through the substrate and air. Mates are attracted to the same plant by pheromones, where they exchange information through mechanical and close-range chemical signals. Plants absorb odor molecules from insects and produce kairomones that can enhance or reduce insect responses to pheromones. Long-range communication between stink bug mates in the field occurs exclusively via pheromones. The species specificity of the sex-pheromones guarantees their success in finding mates in the plant environment. Substrate-borne communication occurs in a narrow frequency range that is tuned to the mechanical properties of the plant. Vibratory signals are transmitted with low attenuation and with altered frequency, amplitude, and temporal characteristics. The frequency sensitivity of the subgenual organ is tuned to the low-frequency resonant properties of the plants. Recognition is encoded in the vibratory signal species- and sex-specific temporal parameters and directionality in the time delay between signals arriving from different directions. The characteristics of behaviorally described multimodal close-range communication on a plant are under-investigated. Studies of neuronal processing of multimodal sensory signals in the stink bug brain are needed to understand how their integration affects behavioral responses.

**Abstract:**

Plants influenced the evolution of plant-dwelling stink bugs’ systems underlying communication with chemical and substrate-borne vibratory signals. Plant volatiles provides cues that increase attractiveness or interfere with the probability of finding a mate in the field. Mechanical properties of herbaceous hosts and associated plants alter the frequency, amplitude, and temporal characteristics of stink bug species and sex-specific vibratory signals. The specificity of pheromone odor tuning has evolved through highly specific odorant receptors located within the receptor membrane. The narrow-band low-frequency characteristics of the signals produced by abdomen vibration and the frequency tuning of the highly sensitive subgenual organ vibration receptors match with filtering properties of the plants enabling optimized communication. A range of less sensitive mechanoreceptors, tuned to lower vibration frequencies, detect signals produced by other mechanisms used at less species-specific levels of communication in a plant environment. Whereas the encoding of frequency-intensity and temporal parameters of stink bug vibratory signals is relatively well investigated at low levels of processing in the ventral nerve cord, processing of this information and its integration with other modalities at higher neuronal levels still needs research attention.

## 1. Introduction

Most insects spend their life in a plant environment. Herbivorous stink bugs of the family Pentatomidae (Heteroptera) represent a well-studied example of how plants determine the evolution of multimodal sexual and mating communication processes at the emitter and receiver sides. Stink bugs or true bugs are the fourth largest family within Heteroptera, and about 3336 species of the most abundant subfamily Pentatominae are distributed worldwide [1,2]. The globally important pest status of many Pentatominae species has been the catalyst for numerous studies on their biology [1], host and associated plants [3], and biorational control techniques to maintain pest populations at sustainable levels [4]. Profound knowledge of the biology of Pentatominae studied in detail in the Neotropics gives the essential basis for the long-term research on group communication processes as the key to population success in a complex and changing environment.

Pentatominae are phytophagous, polyphagous, and solitary species that feed on various plant parts, preferentially on their fruits and seeds [5,6]. The development from egg to adult occurs in Pentatominae on preferred host plants, but in their absence, they show high feeding plasticity from monophagy to oligophagy and polyphagy by moving to spatially associated plants in their environment [3,5]. In the Neotropics, the polyphagous stink bug *Nezara viridula* prefers 29 different host plants belonging to the families Fabaceae (legumes) and Brassicaceae (cabbage, collard, mustard, radish, broccoli, etc.), and shifts to 197 other plant species within 43 families in their absence [7,8,9].

**Figure 1:**
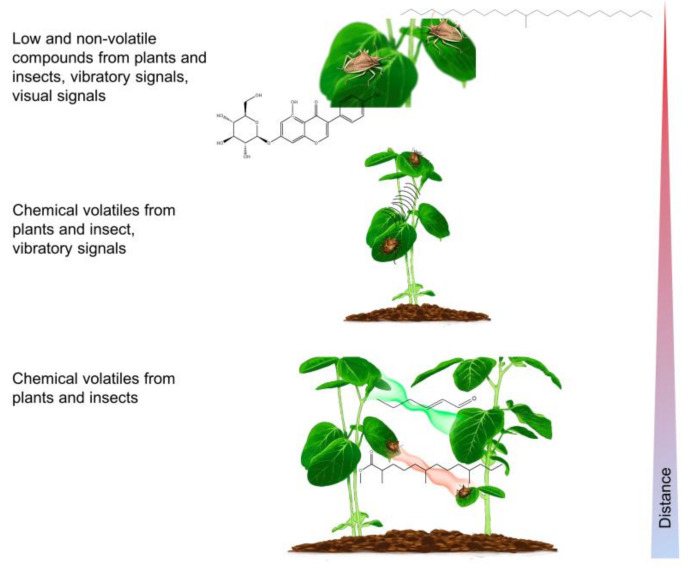
Schematic drawing showing the signals that are produced in plant–stink bug communication at different spatial scales (i.e., distances).

The reproductive biology of stink bugs was recently described and reviewed by Grazia and Schwertner [1]. Females lay eggs on host plants, where nymphs develop into adults over five nymphal stages in 40 to 80 days. The number of generations per year varies from one to seven. The quality of host plants influences life traits in Pentatominae stink bugs [3,10,11,12]. Mating behavior and communication have been described in more than 35 stink bug species of the subfamily Pentatominae [13,14,15]. Stink bugs dispersed in the field congregate on the same plant, attracted by airborne chemical signals, and communicate there during calling, courtship, and rivalry phases by signals of different modalities (Figure 1) [16].

The present review aims to highlight the key role of plants in the multimodal airborne and substrate-borne communication of stink bugs in the field. We describe how plants modify communication signals through their presence and as a transmission medium and how stink bugs match the properties of their emissions with the specific properties of plants to obtain an optimal signal-to-noise ratio and increase the communication distance. Receptor organs with specific morphology and function are described as constitutive elements of the complex multimodal sensory system of the stink bug, which is capable of detecting, encoding, and extracting the information preserved in the parameters of the signals transmitted in the plant environment. Finally, we present mechanisms of central nervous processing of vibratory signals that shape the stink bug’s orientation and recognition behavior on a plant.

## 2. Long-Range Sex Pheromone Communication in Stink Bugs in a Plant Environment

**Figure 2:**
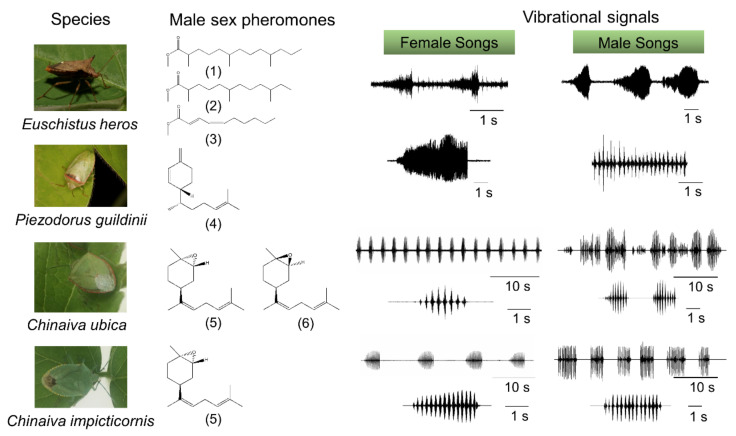
Specificity of chemical and vibrational signals in four Neotropical stink bugs species. Species-specificity of male sex-pheromone: (1) methyl 2,6,10-trimethyltridecanoate, (2) methyl 2,6,10-trimethyldodecanoate, (3) (2E,4Z)-methyl deca-2,4-dienoate, (4) 7*R*-β-sesquiphellandrene, (5) *trans*-(*Z*)-(4*S*) bisabolene epoxide, and (6) *cis*-(*Z*)-(4*S*) bisabolene epoxide. Vibratory signals represent female and male calling signals of each species. Time scales are in seconds (s).

Long-range communication between stink bug mates in the field occurs exclusively via semiochemicals (pheromones) [16,17,18,19]. Stink bugs emit different chemical signals as sex, aggregation, and alarm pheromones [20,21,22]. The pheromones identified to date from species in the subfamily Pentatominae are all produced by males (Figure 2). In Neotropical species, all of these are sex pheromones that attract only females [16], whereas in Nearctic species, such as *Piezodorus hybneri* and *Murgantia histrionica*, the pheromone produced by males attracts both sexes and nymphs, working as an aggregation pheromone [23,24,25]. Pheromones that play an important role in the mating behavior of Pentatomidae are very well described for only 32 species [16,26,27,28]; of these, 14 produce fatty acid derivatives compounds as pheromones, 15 produce sesquiterpenes, and 3 produce a combination of fatty acid and terpene derivatives as pheromone components [16]. Some Pentatomidae pheromones present chemical structures similar to plant compounds or are produced by plants. For example, the sex pheromone of *Tibraca limbativentris* comprises a combination of the two isomers of zingiberenol, (3*S*,6*S*,7*R*) and (3*R*,6*S*,7*R*)-1,1-bisaboladien-3-ols, and two isomers of sesquipiperitol are also present in plants such as rice and Zingiberaceae plants [29,30]. Males of *Piezodorus guildinii* produce, as sex pheromones, the sesquiterpene (7*R*)-sesquiphellandrene, a compound also produced by rice, ginger, sorghum, wheat, and other plants. Other stink bugs produce, as sex pheromones, compounds identified only in insects, such as the fatty-acid-derived pheromones methyl-2,6,10-trimethyltridecanoate produced by males of *Euschistus* *heros* and methyl-(2*E*,4*Z*,6*Z*)-decatrienoate produced by males of *Thyanta perditor* [16]. Two recent studies conducted by Lancaster and collaborators [31,32] showed that two stink bugs, *M. histrionica* and *N. viridula*, produce their pheromones de novo through two different isoprenyl diphosphate synthases, *Mh*IDS-1 and *Nv*IDS; enzymes that function as terpene synthase enzymes (TPs) converting farnesyl diphosphate into their pheromone precursors [31,32]. Therefore, these two stink bugs do not sequestrate compounds from plants or microorganisms as precursors to produce their pheromones; and this can probably be generalized to other stink bugs [16].

Another question that still needs a response is that if the host location by stink bugs could be influenced by plants that produce compounds similar or identical to their sex or aggregation pheromones. It is well known that stink bugs are attracted to volatiles emitted by host plants [33,34], but there is still little information about what the plant volatiles involved in this attraction are [35,36,37]. Second instar nymphs of *Antestiopsis thunbergii* are attracted to anisole, methyl 3-ethyl-4-methylpentanoate, and (5*S*,7*S*)-conophthorin emitted by mature green coffee berries [35]. Another study reported that *Bagrada hilaris* is strongly attracted to a tricyclic diterpene hydrocarbon, brassicadiene, in laboratory bioassays [36,37].

Most studies covering the chemical communication of stink bugs and plants concentrate on tritrophic interactions. Plants produce a vast diversity of volatile compounds, such as terpenes (mono, homo, and sesquiterpenes); for example, linalool, (*E*)-β-caryophyllene, α-(*E*)-bergamotene, β-farnesene, zingiberenol, sesquipiperitol, and (*E*,*E*)-4,8,12-trimethyl-1,3,7,11-tridecatetraene (TMTT), aromatic compounds from the shikimic pathway (for example, methyl salicylate and indole), and green leaf volatiles, such as (*Z*)-3-hexenyl acetate and (*E*)-2-hexenal [38,39,40]. Data on the behavioral or eletroantenographic responses of phytophagous stink bugs to host plant volatiles are summarized in different studies in the literature [35,39,40]. In general, the constitutive plant volatiles are emitted in tiny amounts, and plants enhance the emission of these volatile compounds when injured by herbivory or ovipository damage [33,34,41,42,43]. Plant volatiles can work as kairomones to herbivores, but there is no evidence that plant volatiles influence stink bugs’ pheromone production or enhance their attraction to pheromone traps. Plants injured by stink bugs emit higher levels of volatiles compared to undamaged plants. These herbivory-induced plant volatiles (HIPVs) are less attractive to conspecifics than constitutive plant volatiles, although HIPVs are highly attractive to natural enemies of stink bugs. This non-preference for injured plants may be related to stink bugs avoidance of plants that are likely to be visited by natural enemies. Further studies are necessary to elucidate, for example, whether plants are able to recognize the presence of stink bugs through their pheromone emission with no previous tactile contact [44,45] and whether plants that produce the same or similar stink bug pheromone compounds influence the host plant location.

### Neuronal Basis of the Perception of Semiochemicals in Stink Bugs

Olfactory and gustatory receptors are largely distributed on different parts of the insect body, with most chemoreceptor sensilla located on the flagellum of the antennae, in addition to mechano, thermo, and hygroreceptors [46,47]. In stink bug species, such as *N. viridula* [48], *Cyclopelta siccifolia, Chrysocoris purpurea* [49], *P. guildinii*, *Edessa meditabunda,* and *E. heros* [50], chemosensory sensilla have been described mainly on the filiform type of the flagellum. The higher number of porous sensilla found on the flagellum of *E. heros*, *E. meditabunda,* and *P. guildinii* females as compared to males suggests their implication in sex pheromone detection [50].

Detailed studies on the detection, transduction, and neuronal processing of odor molecules, as recently summarized by Renou and Anton [51] for other insects, have not been, to our knowledge, sufficiently explored in stink bugs. However, odorant-binding proteins (OBPs), which are responsible for the transport of odorant molecules to the membrane-bound olfactory receptors (ORs) in the outer dendrite, have been identified in four stink bug species. A total of 23, 25, and 9 OBPs were found in *E. heros*, *C. ubica,* and *D. melacanthus,* respectively, but their ligands are unknown [52]. In the lychee giant stink bug *Tessarotoma papillosa* olfactory (ORs) and ionotropic receptors (IRs) were identified [53] and for *Halyomorpha halys* 44 OBPs and 17 chemossensory proteins (CSPs) were identified [54].

Hemiptera antennal olfactory receptor neurons project to a well-developed antennal lobe with distinct glomeruli [55,56]. The central olfactory system at higher neuronal levels has been investigated very little [56,57]. Neurobiological studies are needed in the brain to understand the integration of chemosensory information with other multimodal sensory input, provided through different channels during stink bug mating behavior and communication between and on the plants.

## 3. Multimodal Communication on a Plant

**Figure 3:**
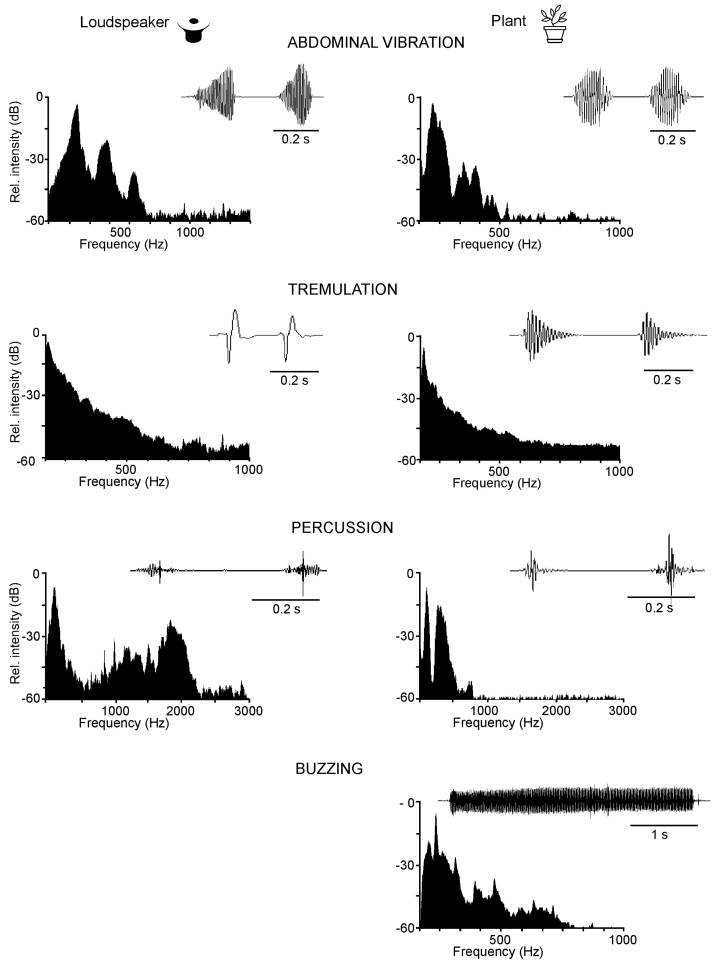
Vibratory signals in stink bugs produced by different mechanisms. Examples of abdominal vibration signals, tremulation and percussion signals of a *P. maculiventris* male, and a buzzing signal produced by an *E. heros* female. The signals recorded from the loudspeaker membrane (**left**), and from the plant (**right**) indicate the influence of the substrate properties on the signal characteristics. Shown are oscillograms of 1–2 pulses or pulse trains with the frequency spectra. New data and new data analysis [64].

Intraspecific stink bug long-range communication with semiochemicals is followed on a plant by the substrate-transmitted component of vibratory signals, produced by different mechanisms (Figure 3), short-range visual signals and cues [58], and contact chemical and mechanical signals [14]. Even though we cannot exclude communication in stink bugs by the air and/or soil-borne components of vibratory signals transmitted between neighboring plants, as shown in many arthropods, such as ants, scorpions, fiddler crabs, and insects [59,60,61,62,63], we will here only treat short-range communication on the same plant. 

### 3.1. Communication during Calling, Courtship and Rivalry

The main part of the information exchange of stink bugs on a plant is via the substrate-borne component of abdominal vibration (AV)-produced signals [65,66,67]. In *N. viridula* and several other stink bugs, communication begins during the calling phase of mating behavior by the female’s emission of calling song signals triggered by pheromones released by conspecific males [66] (Figure 2). Males respond with the calling and courtship signals produced when approaching the calling female [13,14,15]. Female–male duets represent the main line of information exchange that brings them together. The AV signals enable mate finding and recognition [64,68,69,70,71,72]. 

The calling phase ends with the close encounter of mates and transitions into courtship, which is characterized by multimodal communication with AV female and male courtship signals accompanied by visual, chemical, and mechanical contacts (13–15). Calling and courtship communication is inhibited by the rivalry between males or females when two or more of them compete for copulation with a single mate [13,73,74].

**Figure 4:**
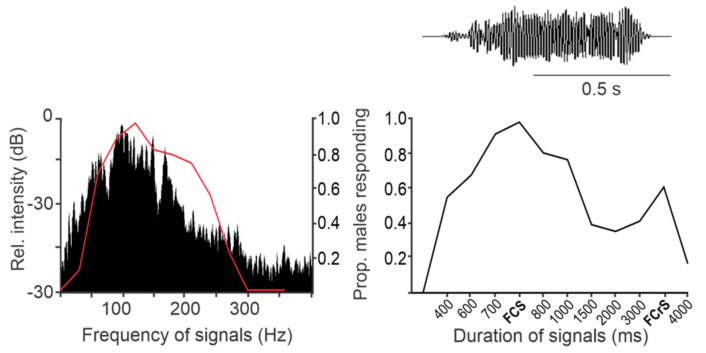
Male preference curves for frequency (**left**) and duration (**right**) compared to spectrum and duration of female calling song (FCS) and female courtship song (FCrS) signals in *N. viridula*. Shown are the frequency spectrum (**left**) and duration (**right**) of a FCS signal characteristic of the mean frequency and duration for the population, with the proportion of males (N = 23, N = 14) responding to synthesized signals varying in dominant frequency (**left**) and duration (**right**), while other signal parameters were kept constant (i.e. at the mean value for the population). New data analysis according to [64].

The AV signals have been described in 36 species of stink bugs as components of the calling, courtship, rivalry, and copulation songs [13]. They share a fundamental (basic) frequency that ranges predominantly from 90 to 120 Hz (Figure 4), with the extreme lowest and highest values measured to date in *N. antennata* (50 Hz, female calling song) and *E. heros* (175 Hz, male courtship song) [75,76]. AV signal amplitudes (expressed in velocity units) range from 0.1 to 1 mm/s when measured on the body of bugs standing on plants [77]. The AV signals express the greatest variety of temporal characteristics that determine their species and sex specificity. The pattern and temporal characteristics of the same song type signals can differ significantly between populations of the same species, as shown, for example, in *N. viridula* from Brazil, Florida, Italy, and Slovenia [78]. Genetic differences between 11 geographically separated populations of *N. viridula* from Europe (Slovenia, France, Greece, Italy, and Madeira), Japan, Guadalupe, Galapagos, California, Brazil, and Botswana have been described by Kavar and co-workers [79]. Several playback experiments confirmed that the duration of song components and the intervals between them mediate recognition and directional information [64,71,72,80].

The pattern of calling, courtship, and rivalry songs reflects their basic function in communication [13]. Longer emission periods of signals with stable temporal parameters characterize the calling song, which directs movement toward the recognized calling mate. More complex courtship songs, supported by signals from other modalities, enhance recognition and motivate mates to copulate. Male rivalry songs of most species studied to date show common characteristics as a short-term production of rapidly repeated shorter and often frequency-modulated (FM) pulses leading to an a-b-a-b alternation that silences one of the rivals [13,14,15]. Female rivalry is more complex and involves prolonged alternation with the rival and differently modified calling signals [73,74]. In *E. heros*, *C. impicticornis,* and *C. ubica* stink bugs, high-amplitude, broad-band, and species and sex-non-specific signals produced by the vibration of lifted wings (buzzing signals) or tremulation of the whole body (tremulatory signals) have been described [59,81]. Buzzing signals precede emission of the AV signals predominantly in the very early phase of mating [81], and tremulation of the whole body has been observed in the context of aggression during rivalry and body positioning prior to copulation [59]. Stink bugs produce low-amplitude percussion signals (also called copulatory signals) by tapping the substrate with their forelegs immediately after establishing copula [13]. The role of species and sex-specific percussion signals is not yet clear. Chemical signals also play an important role in communication on the plant. The male pheromone triggers the emission of the calling song in *N. viridula* females, and playing back the latter increases the amount of male pheromone release, the compounds *cis* and t*rans* (Z)-bisabolene epoxide [58,82]. Recently, a similar response was observed for *E. heros* (Aline Dias, Raul Laumann, Miguel Borges, Maria Carolina Blassioli-Moraes, unpublished data). 

Antennation of the partner’s body provides additional identity information via species and sex-specific cuticular hydrocarbons, as described in *N. viridula*, *C. ubica,* and *C. impicticornis* [83,84]. Guarino et al. [85] demonstrated in *Bagrada hilaris* that males significantly reduced their copulation attempts with females whose bodies were washed with a solvent that extracts cuticular hydrocarbons. The chemical footprints of *N. viridula* (a series of saturated linear hydrocarbons from C19 to C40) walking on the plant remain in the wax layer of the plant [86] and can serve as cues to locate and identify mates on the plant. 

### 3.2. Plants as the Vibratory Signal Transmission Medium

Among various modalities of mechanical signals, insects use substrate-borne vibrations as the most common means of communication on plants or other substrates [87]. Studies in spiders [88], scorpions [61], stink bugs, and many other insect groups [13,14,15,89,90] have described different mechanisms involved during substrate-borne vibratory communication. As a non-inert medium, plants significantly change the characteristics of transmitted vibrations [91]. Michelsen and co-workers [92] showed that cydnid bug and leaf and planthopper vibratory signals are transmitted through different plants as bending waves of low and frequency-dependent propagation velocity, with low attenuation, and non-linear amplitude decrease with the distance. The authors measured the lowest attenuation of around 100 Hz plant-transmitted vibrations. The amplitude of *N. viridula* AV ranged from 0.1 to 1 mm/s when recorded on the body of the bug and on the plant immediately below [77]. The approximately 1 mm/s velocity of *M. histrionica* AV signals recorded from the body differed when recorded on various substrates at a distance of 1 cm from the source from around 0.2 mm/s on the non-resonant loudspeaker membrane to 2.3 mm/s on the London rocket (*Sisymbrium irio)* leaf [77]. The amplitude decreased by 0.06 and 0.1 dB/cm during transmission through the sedge stem, with regularly repeated peaks of amplitude minima (nodes) and maxima (antinodes) [77]. The largest velocity difference between adjacent nodes and antinodes can reach 20 dB. Different amplitude decays were measured for AV signals transmitted through different parts of the leaf. The regular amplitude variation with distance for the *N. viridula* stem-transmitted vibration was described as a consequence of the resonance of the plant [93]. At five centimeters from the source, the velocity of *M. histrionica* play-back signals decreased between 3.6 and 4.6 dB/cm for lamina-transmitted and between 0.4 and 0.8 dB/cm for vein-transmitted vibrations [94]. The amplitude decay of plant-transmitted tremulatory signals differs. The velocity of up to 14 mm/s of the asopine stink bug species *P. maculiventris* decreased by 0.2 to 0.3 dB/cm when transmitted through the *Plumbago* stem, with a rapid decay within the first 18 cm and a steady decrease in velocity over the remainder of the 93 cm stem [95,96]. The transmission properties of percussion signals described in Asopinae [96] and Pentatominae [59] have not yet been studied. The frequency characteristic*s* of the AV and buzzing signals are tuned with the mechanical properties of the stink bug hosts and associated plants [69,81,93]. The spectra of all AV signals recorded to date show unique characteristics: the narrow fundamental frequency peak is around 100 Hz in the majority of species, and the harmonic peaks do not exceed 600 Hz. Frequency modulation (FM) characterizes signal subunits in species, such as *P. lituratus, E. heros,* or *Halyomorpha halis* [97,98,99]. Various experiments have confirmed the low-pass filtering properties of herbaceous plants. Sinusoidal vibrations of frequencies around 100 Hz were less attenuated when transmitted through *Thesium bavarum* [92]. The pure-tone fundamental frequency of 124 Hz changed to 84 Hz when transmitted through a sedge stem [100]. Relatively stable fundamental frequency and higher attenuation of spectral components above 500 Hz have been measured in vibration signals of *N. viridul*a and *M. histrionica,* recorded on different plants and at different distances from the source [77]. The root-dwelling bugs *Scaptocoris carvalhoi* and *S. castanea* (Cydnidae) emit stridulatory signals at the broad 500 Hz dominant frequency, which disappears in the dominant frequency signal around 100 Hz, recorded from the soybean stem above the soil [63]. Bending waves propagate through the rod-like structures of the plant with a frequency-dependent velocity [92]. The distance between the relative amplitude peaks of the fundamental frequency is twice the distance between those of the first harmonic, and consequently, the ratio between the relative amplitudes of the two spectral peaks is different at different distances from the source [77]. The highest signal amplitude coincides with the highest amplitude difference of the spectral peaks, and the lowest signal amplitude was measured at distances where the difference was at its minimum. The different ratio between the amplitude peaks of the fundamental and the first harmonic frequency has a distance-dependent effect on the amplitude modulation (AM) pattern of signals composed of spectrally different or FM subunits. Laboratory experiments are needed to test the hypothesis that the perception of these differences can be used by insects to obtain information on distance and direction to the source on the plant. Similar spectral changes have been recently described for plant-transmitted stink bug buzzing signals whose fundamental frequency varies in the same range as that of AV signals [81]. However, differences should be expected for tremulatory signals, whose spectral characteristics resemble those measured for leaf vibrations induced by falling raindrops, balls, or landing parasitoids [101]. The plant resonance contributes to the position of the dominant peak around and below 10 Hz of the long regular phase of the tremulatory signal, which follows the short up to 25 kHz irregular phase at the beginning of the signal. Different amplitude ratios of the low-frequency spectral peaks of the tremulatory signals of the predatory bug *P. maculiventris* were measured on plumbago or bean at different distances from the source [95]. The frequency components above 500 Hz of the percussion signals of *Lygus rugulipennis* (Heteroptera: Miridae) confirm the low-pass filtering properties of plants [102].

The temporal characteristics of vibratory signals are altered as they are transmitted along the plant [92]. Miklas and co-workers [103] demonstrated that in *N. viridula*, increasing the duration and the consequent decrease in the interval between the components of the signals transmitted by the plant prevents recognition of the song. The calling song signals (pulses) of females elicit responses from males [78], and the rival song signals (pulse trains) of females inhibit them [14,74]. Males discriminate both signals of similar duration and repetition time when played on the non-resonant substrate. On the plant, males respond equally to both types of signals, suggesting that a longer duration of rapidly repeated train pulses fuses them to the point that males recognize them as the unique calling song pulses. The duration and intervals of overlapping signals change when insects sing simultaneously. De Groot and co-workers showed that overlapping *N. viridul*a female calling song signals altered their species-specific temporal structure and reduced conspecific male responses [104].

### 3.3. The Effect of Noise on Signal Transmission on Plants

Efficient communication requires co-evolution of signals, signaling behavior, and sensory systems, which are influenced by properties of the transmission medium and environment noise [105,106]. Stink bug band-pass communication tuned to the transmission medium (plants) and the highly adapted sensory system (see below) has the advantage of filtering out noise from frequencies below and above the band limits, but also narrows the range of species-specificity of signaling to differences in signal temporal, pattern, and AM properties. Furthermore, noise with similar frequency characteristics has a pronounced effect on signal characteristics through interference, as has been shown, for example, in overlapping signals from *E. heros* [98] (see below).

Wind and falling water drops represent the main source of noise in the field [87,101,107,108,109,110,111,112]. Spectra of wind-vibrated apple leaves are characterized by broad frequency bands up to 25 kHz with a dominant frequency peak from 10 to 14 Hz and amplitudes reaching 130 mm/s for high wind speed [101]. High amplitude signals generated by falling water drops are characterized by up to 29 ms broadband irregular phase followed by an up to 10 Hz regular phase with a half-life of 163 ± 37 ms (N = 12) [101].

The main energy of stink bug AV signals and the peak velocity thresholds of the most sensitive receptor organs of the group (see below) do not match those characteristic of wind-generated vibrations. Nevertheless, Dias and co-workers observed significantly fewer pairings and copulations in laboratory experiments when *E. heros* stink bugs were exposed to airflow or rain-generated noise [74]. Direct field experiments are needed to confirm or reject the hypothesis that low-velocity wind does not significantly reduce vibrational communication on a plant.

Polajnar and Čokl studied the responses of *N. viridula* males and females to 80 to 115 Hz pure-tone background vibration at velocities around 1 mm/s [113]. One hundred Hz background noise did not significantly reduce the number of males searching the calling female, nor did the average time spend doing so. However, females stopped calling, decreased the repetition rate of their emissions, or changed calling to rival singing. Furthermore, females changed the fundamental frequency of their calling song to increase the difference to the frequency of the pure-tone background vibration. 

Recently, Laumann and co-workers confirmed the different effects of pure tone noise on male and female communication, reproductive behavior, and long-term reproductive success in *E. heros* [114]. Continuous background vibration at 20 Hz had no effect, but 75 to 200 Hz pure tones significantly delayed singing, searching behavior and consequently decreased the number of copulating pairs. Twenty-four hours of a 125 Hz pure tone vibration on plants reduced the mating frequency of stink bugs by 25% and decreased female fecundity and fertility [74].

By comparison, Spezia and co-workers described a positive impact of noise on the male *N. viridula* responses to female calling [115]. The authors stimulated males with pre-recorded female calling song signals applied to the tip of a branch of a Y-shaped wooden dummy plant and measured their directional movement towards the ipsi or contralateral branch. Subthreshold female calling song signals in the presence of Gaussian “white noise” with flat spectral characteristics up to 22 kHz activated males to search for the source. A non-monotonic behavior was observed at increased noise levels: responses increased to the optimal level and decreased at even higher noise intensities. The authors considered the non-monotonic behavior to be the signature of stochastic threshold resonance.

The active response to changing parameters of overlapping signals of similar frequency has been described in detail in *E. heros* [98]. Female signals overlapped male responses until males and females matched their repetition rate and duration to silent periods between mate’s emissions. Masked female and male calling signals with similar fundamental frequencies around 110 Hz induced interference that significantly changed the overlapping region into a sequence of regularly repeated and fused pulses, with amplitudes and durations increasing as the difference between the fundamental frequencies of the overlapping vibrations decreased. To avoid interference, females and males adapted the repetition rate of their calls and changed the fundamental frequency to increase the difference between them. Recent experiments in *C. impicticornis*, *C. ubica,* and *N. viridula* confirmed the role of rival songs as an inhibitor of simultaneous singing by multiple mates to prevent masking of the signals and consequent reduction in their information value [13,14,15].

## 4. Detection of Vibratory Signals on a Plant

Behavioral responses to vibratory signals are triggered by the integration and processing of information provided by different unimodal receptors that act as constitutive elements of the system, recognizing and encoding the amplitude, frequency, and temporal characteristics of the sensory input. Behavior during stink bug close-range vibrational courtship has been described in several species, but the neuronal mechanisms underlying these processes remain rather scarcely investigated, only including a few species and focusing largely on the receiver site [13].

### 4.1. Vibration Receptor Organs

A number of mechanoreceptors with different sensitivities and selectivities are involved in plant-borne vibratory communication [13,14,15]. The specialized subgenual chordotonal organ of stink bugs is the most sensitive in the frequency range characteristic of AV signals and resonant properties of plants. The chordotonal organs of the leg joints and antennae, in addition to the campaniform sensilla, detect lower frequency components of AV, buzzing, and tremulatory signals. Chordotonal organs, recently described in the stink bug body, are indirectly involved in vibrational communication [116]. The potential role of hair sensilla (trichobotria), which are highly sensitive to the movement of air particles generated by the vibration of the body or some of its parts, remains to be confirmed experimentally in stink bugs.

The subgenual organ of *N. viridula* reflects the highest adaptation of structure and function to detect the frequency, amplitude, and temporal characteristics of AV signals transmitted through plants [117,118]. The organ, which has been described as having two scolopidia (each with one sensory cell) in *N. viridula* [119] and in the brown-winged bug *Plautuia stali* [116], extends longitudinally within the tibial hemolymph channel of each leg. The body is fixed to its inner wall close to the femur–tibia joint, and distally, the flag-like ligament, consisting of two cap and attachment cells, fix it at various points in the vicinity of the joint with the tarsi. 

Mechanical force is transmitted to the membrane of both receptor cells via the cuticle and the wall of the hemolymph channel. A significantly prolonged response of sensory cells to stimuli around 200 Hz, with characteristic resonance spike rate decay, indicates the additional input from the flag-like ligament oscillating within the hemolymph channel. Furthermore, we cannot exclude input from the vibrating air in the closely apposed leg trachea. Both sensory cells act as acceleration receivers below and as displacement receivers above the frequency of highest sensitivity, recorded at around 200 Hz (threshold ca. 0.01 mm/s) in one cell and between 700 and 1000 Hz (threshold ca. 0.002 mm/s) in the other [117].

The threshold sensitivity of both cells around 100 Hz ranges 20 to 40 dB below the amplitudes of the AV input signals recorded on the body of investigated stink bug species. The increased sensitivity of both cells at frequencies around 200 Hz enables detection of the first harmonic frequency, and the high sensitivity above 500 Hz of one sensory cell of the subgenual organ detects higher frequency components of buzzing signals.

**Figure 5:**
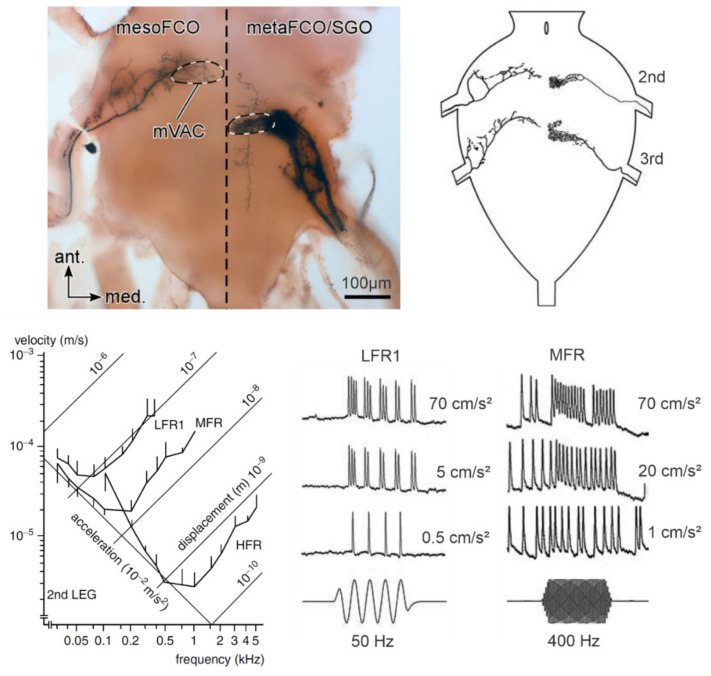
Central projections and physiological responses of vibratory receptor neurons in stink bugs. The micrograph shows anterograde staining of a single mesothoracic FCO (**left**) and a group of metathoracic FCO/SGO sensory neurons (**right**) terminating in the central ganglion. The dashed line outlines the area of the medial ventral association center (mVAC). The dashed vertical line shows the midline of the ganglion (adapted from Nishino et al. 2016, with permission from Springer Nature). **Top right** are combined drawings of intracellularly stained sensory neurons of *N. viridula* morphologically corresponding to FCO (**left**) and SGO (**right**) afferents. **Bottom right** recordings are example traces of responses from the neurons projecting to the mesothoracic segment (second) of the central ganglion. The stimuli are 100 ms long sinusoidal vibrations of the indicated frequencies and intensities. The two neurons physiologically correspond to the low-frequency (LFR1; FCO neuron) and the middle-frequency (MFR; SGO neuron) receptor types. The diagram shows mean threshold curves of the middle leg LFR1, MFR, and high-frequency (HFR) receptors physiologically characterized in *N. viridula* (adapted from Čokl et al. 2006 [63], with permission from Springer Nature, and from [124], with permission from Taylor&Francis Group).

Zorović described intracellularly recorded responses of the subgenual organ receptor cells of *N. viridula* (Figure 5). One cell responded tonically in the range of 50 to 700 Hz, with the best sensitivity between 100 and 150 Hz [118]. The other cell with the highest sensitivity between 300 and 500 Hz was spontaneously active and showed presynaptic inhibition to low-amplitude signals and frequencies below 100 Hz. Presynaptic inhibition regulates sensory input in crickets and stick insects [120,121,122,123], and Zorović suggests that the presynaptic inhibition of neurotransmitter releases from primary sensory interneurons decreases the sensitivity of receptor cells to prevent their desensitization during the production of AV signals [118].

The leg joint chordotonal organs of the stink bug *N. viridula* have a reduced number of scolopidia [116,119] compared to other insect groups [125]. The FCO is the most complex and consists of the proximal condensed scoloparium with eight and the distal dispersed scoloparium with four scolopidia, each containing two sensory cells. The organ is attached proximally to the antero-dorsal region of the cuticle and distally to the *m. levator tibiae* and tibial apodeme. 

A detailed comparative study of the morphology and innervation of the FCO was recently described in *P. stali* (Pentatomidae) and in the shield bug *Parastrachia japonensis* (Cydnidae) [116]. The authors described the ventral and dorsal scoloparia attached distally by the ventral and dorsal ligaments, similar to those in lacewings [126]. The ventral ligament is attached to the dorsal side of the apodeme extensor and the dorsal ligament to the postero-central region of the accessory extensor muscle. The sensory nerve, which branches dorsally from the main leg nerve, innervates both the FCO and the subgenual organ of *P. stali* and *P. japonensis* [116]. The subgenual organ, hair sensilla, and campaniform sensilla located in and on the proximal part of the tibia are innervated by a nerve branching from the ventral region of the FCO scoloparia.

Recordings from individual sensory neurons during vibration of the legs of *N. viridula* revealed a group of low-frequency receptor (LFR) neurons characterized by highest frequency sensitivity between 40 and 75 Hz, a phase-coupled response pattern below 150 Hz, response latencies between 10 and 13 ms to 100 Hz vibratory stimuli, and thresholds following the line of equal displacement values around 0.1 μm [117,118] (Figure 5). The three basic types of LFR neurons are distinguished by their phase-locked responses, which occur either along or at the peak amplitude of upward or downward leg movement. The number of action potentials per phase increases with increasing displacement. The exact peripheral location of individual LFR cells has not yet been determined. Phase-coupled responses of the LFR neurons allow precise frequency coding below 150 Hz. The fundamental frequency of the AV signals studied from the genera *Chinavia*, *Chlorochroa*, *Murgantia*, *Nezara*, and many other stink bug species is below 100 Hz [13], with an extremely low value around 50 Hz measured in the calling song pulses of the female of *N. antennata* [76]. Phase-locked responses precisely encode the signal FM, characteristic of species such as *Piezodorus lituratus* [127], *N. viridula* [14,78], and *E. heros* [98]. 

Antennation of the plant has been observed in male stink bugs during their approach to the calling female and between mates during the terminal phase of courtship. In the antennae of *N. viridula*, Jeram and Pabst described the Johnston’s organ with 45 amphinematic scolopidia (each with three sensory and enveloping cells) arranged around the distal pedicellite, and the central chordotonal organ composed of seven mononematic scolopidia (each with one or two sensory cells) attached to the same antennal joint [128]. Recording the responses of antennal mechanoreceptors in the frequency range between 30 and 140 Hz revealed the best sensitivity with a threshold around 1 mm/s for vibrations in the longitudinal direction between 40 and 60 Hz in one type and at 30 Hz in another [129]. The threshold sensitivity of antennal mechanoreceptors, expressed either as displacement or velocity, is about 20 dB lower compared to similarly-tuned LFR neurons. Nevertheless, AV signals around 100 Hz with an amplitude of more than one mm/s were recorded on the stems at the antinode positions, and tremulatory signals reach amplitudes of 20 to 40 dB above the thresholds of the antennal chordotonal organs.

The anatomy and innervation of the abdominal chordotonal organs of stink bugs have recently been described [116]. The tymbal nerve (TyN) innervates the muscles attached to the fused first and second abdominal tergits (“tymbal”) [116]. This nerve is homologous to the tensor nerve in cicadas [130] and to the tympanic nerve in locusts [131]. In *N. viridula*, Amon described a phase-locked one-to-one relationship between the action potentials of the TyN motor neuron, the EMG of the muscles attached to the dorsal plate (tymbal), and the induced vibrations of the substrate [66]. The TyN of *P. stali* also includes sensory neurons innervating various exteroreceptors and the chordotonal organ associated with the tymbal muscles. The chordotonal organ, with four sensory cells attached to the ventral ridge between the metathorax and abdominal sternites, is attached distally to the tymbal muscle by the ligament on the surface of the adipose tissue. The authors also identified and described the pleural group of chordotonal organs located in the pleural fold between the tergites and sternites and the ventral group in the region of the abdominal sternites. The sensory cell of each pleural chordotonal organ is attached by the ligament to the fat body beneath the endocuticle, and the ligaments of the ventral chordotonal organs (each with one sensory cell) posteriorly attach to the surface of the fat body. 

Hair sensilla (trichobotria) detect air movements around the body [132]. The role of trichobotria, located on the stink bug abdominal sterna in nymphs and adults, has not been experimentally confirmed during mating behavior, but the functional properties of trichobotria in the fire bug, *Pyrrhocoris apterus,* show high sensitivity for displacement [133,134] that may be caused by air particle movement in the near field conditions by stink bug production of tremulatory and/or buzzing signals.

### 4.2. Central Projections of the Vibration Receptor Neurons

The medial ventral association center (mVAC) is the extensive mechanosensory neuropile, located bilaterally in all thoracic neuromeres, where axons from leg, body, and antennal chordotonal organs finally terminate and partially overlap [116] (Figure 5).

Axons of four “tymbal” chordotonal organ sensory cells arborize in the mVAC in the meta and mesothoracic neuromeres of the central ganglion and in the prothoracic ganglion; finally, they project with two of them to the mechanosensory and motor centers of the brain [55,116]. Two TyN sensory cells also send short collaterals posteriorly to the abdominal neuromeres of the central ganglion. The extensive arborizations within the mVAC and lack of projections to the motor control center in the dorsolateral neuropiles suggest that the “tymbal” chordotonal organ is not involved in direct control of muscles but provides sensory input to the complex peripheral mechanosensory information processing that occurs in the mVAC. 

Nishino and co-workers suggest that the association of the stink bug’s chordotonal organs with tissues (tendons, muscles, fat bodies, and septa) of various resonant characteristics determines their frequency filtering properties [116]. The pleural chordotonal organ, in close association with the tracheal muscle, and the tymbal chordotonal organ, in close association with the “tymbal” muscles, probably monitor muscle movements. 

Zorović first described single central projections and final arborizations of the receptor cells of the subgenual and joint chordotonal organ in *N. viridula* [118] (Figure 5). Axons of the leg chordotonal organs of *N. viridula* terminate ipsilaterally in the prothoracic and corresponding central ganglion neuromeres [118]. Dense terminal arborizations of the sensory cells of the subgenual organ and the joint chordotonal organs with characteristic lateral side branches are located in the corresponding segmental mVAC neuropile regions. Axons of Johnston’s and central organ sensory cells run within both antennal nerves and descend to the abdominal neuromeres of the central ganglion, with side branches projecting to mechanosensory and motor centers in the brain, the prothoracic ganglion, and thoracic neuromeres of the central ganglion [116]. The projections into the mVAC neuropiles suggest co-processing of complex vibratory information, in addition to the sensory input from the abdominal, tymbal, and leg chordotonal organs [116].

The sensory cells of the abdominal chordotonal organs are characterized by extensive intersegmental and local arborizations [116]. The four pleural chordotonal organ sensory cells finally terminate with one of them in the central ganglion, two others in the prothoracic ganglion, and with the fourth in the antennal mechanosensory and motor center in the brain. The six to eight sensory cells of the ventral chordotonal organ send collaterals to the lateral association center and to the mVAC of the metathoracic neuromere of the central ganglion. Chordotonal organs of both groups also send short collaterals to abdominal neuromeres. Axons of antennal mechanoreceptors and “tymbal” chordotonal organs finally terminate with partially overlapping arborizations in neuropiles in the brain, subesophageal, and prothoracic ganglion, and various neuromeres of the thoracic and abdominal parts of the central ganglion. 

The terminal arborizations of the joint chordotonal organs, subgenual organ, Johnston’s organ, and central antennal organ partially overlap, suggesting convergence and coprocessing of plant-borne vibratory information in the mVAC. Nishino and co-workers conclude that chordotonal organs distributed throughout the body may intersegmentally coordinate the production of AV signals by integrating sensory information to generate motor patterns that control the action of “tymbal” muscles [116]. Further neurobiological studies are needed to confirm this hypothesis.

## 5. Processing of Vibratory Input in the Ventral Nerve Cord

Investigations of the neuronal basis of stink bug behavior during communication on the plant have been limited to studies of the vibratory sensory system and the underlying central neuronal network at low levels of processing in the ventral nerve cord [70,135]. Data on neuronal processing of multimodal sensory inputs at higher levels in the brain are lacking.

### 5.1. Frequency and Time Pattern Coding in Ventral Nerve Cord Neurons

Morphology and physiology of four main morphological groups of ventral cord interneurons responding to leg vibration have been described in the stink bug *N. viridula* [70,135]. The complex response pattern of some interneuron types reflects the combination of excitatory and inhibitory inputs that have also been described at the receptor level (see above). 

The first group of interneurons consists of five distinct types, characterized by a cell body in the metathoracic neuromere of the central ganglion (CG) and an axon ascending contralaterally (AC) towards the brain (CG-AC-1 to CG-AC-5). Morphologically distinct types with different response patterns show the highest sensitivity below 100 Hz (CG-AC-1), around 200 Hz (CG-AC-2 and CG-AC-5), 300 Hz (CG-AC-3), and 500 Hz (CG-AC-4). The responses of CG-AC-2 and CG-AC-5 reflect inhibitory inputs at higher intensities in the frequency range below 200 and 300 Hz, respectively. Preliminary data on the likely homolog of CG-AC-1 and CG-AC-5 neurons in the brown marmorated stink bug (*H. halys*) suggest a high degree of conservation of the ventral cord neuronal vibration processing network in the Pentatomidae [136]. 

The second group (CG-AB) is represented by the unpaired median (DUM) interneuron, located with the cell body close to the midline of the mesothoracic neuromere. The neuron has a bilaterally symmetrical structure, including two axons that ascend at least to the prothoracic ganglion. DUM neurons are a large class of local but also intersegmentally projecting neurons described, among others, at low levels of auditory and vibratory processing in Orthoptera [137,138,139] and fruit flies [140], and in honeybees in the context of associative learning [141]. DUM neurons were best investigated in the auditory system of bushcrickets, where they are mostly inhibitory elements and appear to be crucial in frequency and temporal processing [142]. Such a function as inhibitory elements may be generally presumed also for DUM neurons in the insect vibratory system [143]. The DUM neuron example from *N. viridula* is a spontaneously active unit with the best sensitivity below 200 Hz (i.e., in the frequency range of conspecific signals), which receives mixed input with inhibition that precedes the excitatory response [135]. Such preceding inhibition suggests the putative importance of this neuron (also) in the processing of directional vibratory cues, which have to be encoded with a high temporal fidelity [143]. 

Three types of *N. viridula* local interneurons (CG-L) are restricted to the thoracic part of the central ganglion. The spontaneously active neuron CG-L-1 responds to stimulation by inhibition and the other two (CG-L-2, CG-L-3) by phasic tonic excitation in the frequency range below 200 Hz. 

The fourth group is represented by the interneuron, which is localized in the prothoracic ganglion and whose axon descends contralaterally into the meso and metathoracic neuromeres of the central ganglion. The vibratory PT-DC responds below 200 Hz, with the highest sensitivity at 70 Hz and tonic responses at highest amplitudes of 100 Hz stimuli. 

In *N. viridula*, Prešern et al. described the neuronal activity of eleven ventral nerve cord interneurons as sensitive to the time delay between stimulation of the selected legs [70]. Four of the six stained interneurons were clearly different from those previously identified [135]. Three of these intersegmental neurons have local dendritic (input) branches crossing the midline within the same neuromere, and axons running at the soma contralateral site in three of them and additionally ipsilaterally in one of them.

### 5.2. Neuronal Basis of Vibrational Directionality and Mate Recognition on a Plant

The ability to localize the source of vibration on a plant has been demonstrated in *N. viridula* males during their approach to the calling female [68,69]. The searching male stops at a crossing, spreads his legs over different branches, and waits for female vibratory signals to trigger and direct his further movement toward the signal source.

The neuronal basis of vibrational directionality has been extensively studied in the wandering spider *C. salei* [144,145,146]. The authors described vibratory interneurons in the subesophageal ganglion that respond to differences in amplitude (ΔA) or time (Δt) of the arrival of the vibratory signal at different legs in correlation with the directional behavior of the spider. In *Locusta migratoria*, a time delay of one to three ms between stimulation of different legs produces a directional response in the higher-order bimodal neurons tested [147]. 

**Figure 6:**
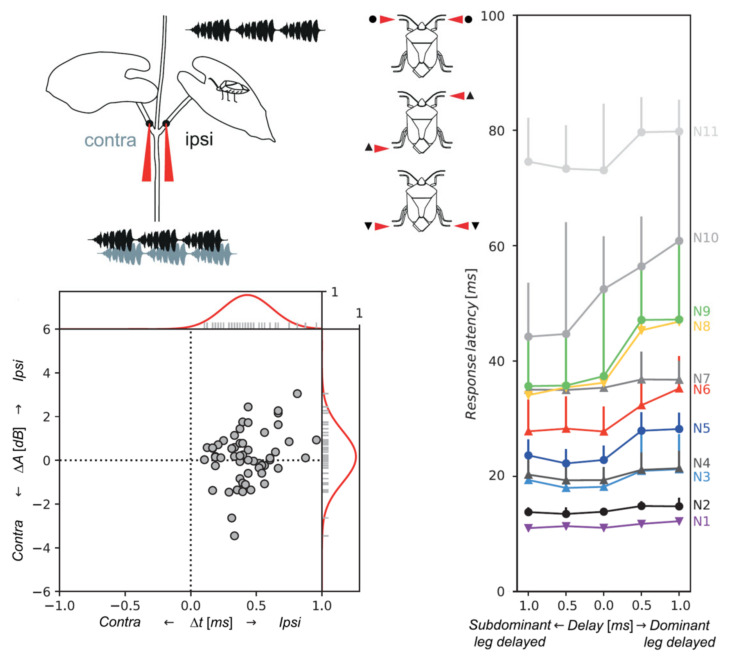
Directional vibratory cues and information coding in the central nervous system of *N. viridula*. Schematic representation of the setup used to measure the transmission of the female’s calling signals across the stem-stalk node of a plant, a relevant decision point for the searching male (**top left**). The signals emitted by the female on a leaf were measured at the indicated points (red) on the same (ipsi) and the opposite petiole (contra) using two laser vibrometers. **Bottom left** is the distribution of time differences (Δt) and RMS amplitude difference (ΔA) of the vibration signals (n = 55) recorded simultaneously at the two points. Values below 0 on the *y*-axis indicate signals with a higher amplitude on the contralateral stalk. Only the difference in arrival time of signals as they reach the male legs straddling the crossing is a reliable directional cue to the calling female. The diagram on the **right** shows the effect of a time delay in the onset of leg stimulation on the response latency of eleven thoracic vibratory interneurons of *N. viridula*. The symbols used indicate the applied configuration of leg stimulation, which is schematized on the **left** of the diagram. The responses show directional sensitivity (i.e., they depend on the side stimulated first). Dominant is the side with the strongest impact (Adapted from Prešern et al., 2018, [70] with permission).

In a recent study on vibrational directionality in the stink bug *N. viridula*, Prešern and co-workers measured a Δt as low as 0.5 ms to elicit changes in thoracic interneuron activity [70] (Figure 6). The mean Δt of 0.41 ± 0.16 ms was measured for simultaneously recorded naturally emitted female (N = 11) calling song signals at points one centimeter apart on opposite leaf stalks. The mean ΔA of 0.16 ± 0.91 dB (N = 11) was measured under the same experimental conditions; however, the authors often detected higher amplitude on the stalk leading away from the calling female. Time delays around 0.41 ms are above the behaviorally determined threshold and correspond to a propagation velocity of about 24 m/s at a distance of 1 cm. This velocity is in the range of values measured for 100 Hz vibrations transmitted along agave and banana plants [148,149], and about that of 100 Hz stem-transmitted dispersive waves induced in the plant by a falling ball [101]. 

A difference of a few dB on bean junctions between amplitudes of natural and artificial signals arriving at ipsi and contralateral legs, with non-constant higher values at the branch leading to the calling female [70], cannot be reliably encoded by intensity-response relations as described for the *N. viridula* receptor and ventral cord vibratory interneurons [118]. Nevertheless, we cannot rule out that ΔA provides the informational cue in the case of its difference between vibratory signals reflected at different distances from the junction. Furthermore, signals received at shorter distances from the source are less altered by transmission than those arriving from more distant reflection points.

The neuronal basis of song pattern recognition in insects has been studied primarily in Orthoptera [150,151,152,153,154,155,156,157]. In these insects, acoustic sensory input is provided by a pair of tympanal ears, and the vibrosensory system in all six legs contributes additional information to the brain via bimodal auditory–vibratory interneurons [158,159]. Behavioral experiments proved that stink bugs recognize mates based on temporal features of plant-borne AV signals [94,95,96]. In *N. viridula,* Zorović confirmed that temporal filtering is based on the selectivity of interneurons for short pulse durations, such as those characteristic of the calling song signals of males of the species [160]. The four ascending interneuron types tested showed no selectivity for pulse durations above 2000 ms, typical of complex pulse trains of male courtship song, and for a constant period or duty cycle, typical of the species’ female calling song. 

Intracellular recordings in some CG-AC neurons revealed three to four regularly repeated post-stimulus oscillations of the membrane potential following the 100 and 300 ms long vibratory stimuli. The 1500 ms stimulus induced only a single post-stimulus increase in the membrane potential of a larger amplitude. The author suggests that the repetition rate of post-stimulus oscillations reflects the temporal characteristics of male calling songs and represents the potential resonance-based mechanism for signal recognition. Neuronal resonance has also been previously observed in the cricket *Telleogryllus oceanicus* [161] and the katydid *Tettigonia cantans* [162].

Neurobiological studies of higher-order interneurons in the brain of stink bugs are necessary to understand the selectivity of behavioral responses triggered by multimodal sensory input.

## 6. Concluding Remarks

Phytophagous stink bugs have evolved various mechanisms to optimize communication in a plant environment through air and substrates. Plant volatiles are used by stink bugs for host location and their chemical communication during mating occurs in the complex plant environment. Mechanical properties of plants alter the frequency, amplitude, and temporal characteristics of substrate-borne vibratory communication signals produced by abdomen vibration at high species and sex-specific level, and of signals produced at low specificity level by the vibration of lifted wings, tremulation of the whole body, and percussion with front legs on the plant’s surface. The specific adaptation of the stink bug mechanosensory system to mechanical characteristics of plants and parameters of abdomen vibration-produced signals is expressed in the morphology of the subgenual organ. The organ’s best sensitivity ranges around the group-specific spectral properties of the vibration signals that are tuned with the frequency low-pass mechanical characteristics of their host and associated plants. Temporal parameters of plant-borne vibratory signals provide species and sex-specificity during the calling and courtship phase of mating behavior. Directionality is mediated by the time delay between vibratory signals arriving from different directions on spatially positioned legs on the plant. Short-range visual and contact chemical and mechanical signals have been observed prior to copulation. Neuronal processing of stink bug leg and antennal mechanoreceptor sensory input has been investigated at the receptor and the ventral cord neuronal level. Further studies are needed in stink bugs to understand the interaction and processing of the multimodal sensory input at higher neuronal levels. 

## Data Availability

Data can be made available to anyone interested by contacting the corresponding author.
